# Cognitive Training Combined with Multifocal tDCS over the Reading Network Improves Reading Performance: A Case of Severe Dyslexia

**DOI:** 10.3390/jcm14165671

**Published:** 2025-08-11

**Authors:** Gloria Di Filippo, Marika Bonuomo, Martina Ravizza, Andrea Velardi, Rinaldo Livio Perri

**Affiliations:** Department of Economic, Psychological, Communication, Educational, and Motor Sciences, Niccolò Cusano University, 00166 Rome, Italy; marika.bonuomo@unicusano.it (M.B.); martina.ravizza@unicusano.it (M.R.); andrea.velardi@unicusano.it (A.V.); rinaldo.perri@unicusano.it (R.L.P.)

**Keywords:** tDCS, dyslexia, neurostimulation, reading, specific learning disorders

## Abstract

**Background:** Developmental dyslexia (DD) is the most common form of specific learning disorders (SLDs). From a neurocognitive point of view, dyslexic reading is associated with atypical neurofunctional patterns in the left hemisphere, mainly in the posterior areas linked to lexical access and phonological processing. Nowadays, rehabilitation treatments do not aim to fix the disorder but rather improve adaptive skills. On the other hand, the transcranial direct current stimulation (tDCS) has recently gained popularity in this field. In fact, a few studies have documented enhanced accuracy and speed after the tDCS over the parietal cortex, although the results were mainly limited to non-word reading. **Methods:** We conducted a single-case study employing an innovative multifocal eight-channel tDCS aimed at increasing the reading network activity in the left hemisphere and inhibiting the contralateral areas. The participant was a 9-year-old boy with a diagnosis of severe mixed-type specific learning disorder. The high-definition multifocal tDCS was administered over key areas of the frontal, temporal, parietal, and occipital lobes (four 3.14 cm^2^ electrodes per hemisphere) in conjunction with tachistoscope training over a span of 10 weeks, with three sessions per week for a total of thirty sessions. Standardized assessments of reading were carried out at the beginning, at the end of the treatment, and at one- and six-month follow-up. **Results:** The treatment led to a 77% improvement in the accuracy of passage reading and an 83% improvement in the reading of high-frequency short words, with stable results at the 1- and 6-month follow-up. By contrast, in line with the severity of the disorder, there were only slight improvements in the speed parameter. **Conclusions:** This is the first study to document such remarkable improvements in reading in a case of severe SLD: if confirmed, these promising findings could pave the way for an effective, non-invasive rehabilitation for SLDs using multifocal tDCS. However, future studies are needed to overcome the limitations of single-case studies, such as the lack of control conditions and quantifiable analysis.

## 1. Introduction

Specific learning disorders (SLDs) are neurodevelopmental conditions characterized by persistent difficulties in the acquisition of academic skills, which typically manifest in reading, writing, and mathematics. These disorders arise during the developmental period and continue throughout life. The severity of SLD varies with age, environmental demands, and contextual factors such as family, school, and work. This means that varying degrees of individual adaptation are required. Within this conceptual frame of reference, it would be misleading to assume that the treatments are aimed at the remission of the disorder. On the contrary, it is reasonable to expect a reduction in severity in terms of deviation from normative parameters and an improvement in adaptation compared to no treatment. Over the years, rehabilitation interventions have focused on various rehabilitation strategies, such as phonological training, training on grapheme–phoneme correspondence, decoding strategies, reading fluency, and the use of coloured reading lenses [for a review, see [1]] or even training on cognitive domains such as attention and working memory [2,3,4]. However, none of these methods showed truly significant results, considering that the average annual increase in reading speed is 0.3 syll/s [5].

A number of interventions have combined phonological–metaphonological training [6,7,8,9,10,11,12], offering children with learning disabilities a series of reinforcement activities aimed at improving metaphonological, reading, writing, and text comprehension skills. Over the past decade, the use of transcranial direct current stimulation (tDCS) has also been tested in the rehabilitation of SLDs. tDCS is a non-invasive brain stimulation approach aimed at modulating cortical excitability through the administration of weak electric currents over the scalp. While the use of tDCS has been supported for the treatment of various clinical conditions in adults, its use in developmental age has only recently been explored [for a review, see [13]]. As for the applications in adults, tDCS in pediatrics has been shown to be safe and to offer a considerable opportunity during the golden period of neural network restoration [14].

tDCS applications for SLDs were promising, with general reports of improvements in reading high-frequency words and pseudo-words [15,16,17]. Most studies in this field have mainly used anodal stimulation over the temporo-parietal cortex (TPC) of the left hemisphere (P7, TP7, or P07 sites of the 10–20 system) with the return electrode over the contralateral site. In particular, Costanzo et al. [18] first reported changes in reading after a single session of tDCS in children and adolescents with dyslexia, documenting a reduction in errors on low-frequency words and faster reading speed on pseudo-words. Similar results were obtained in subsequent studies using the same montage when tDCS (left/right TPC) was combined with cognitive training [15,16,17,19], while Mirahadi et al. [20] documented improvements in pseudo-word reading and phonological awareness in a group of adolescents after fifteen sessions of inferotemporal stimulation (T3/T4). In the sham-controlled study by Costanzo et al. [15], tDCS was combined with cognitive training in children with SLD. They placed the anode over the left temporal–parietal region (between P7 and TP7) and the cathode over the right homologue (between P8 and TP8). The active group showed significant reductions in errors for low-frequency words, as well as improvements in the reading speed of non-words, with stable effects at one-month follow-up. Heth and Lavidor [21] observed significant improvements in the reading speed and the RAN test (rapid automatized naming of numbers and letters) in adults with dyslexia after five sessions of active tDCS (anode over the left V5 area and cathode over the right orbitofrontal cortex) compared to the sham condition. Similarly, Rahimi et al. [22] reported immediate effects of tDCS in children with dyslexia on tasks involving auditory temporal resolution, speech-in-noise perception, auditory–verbal memory, and reading efficiency (speed and accuracy on texts, low-frequency words, and pseudo-words). It is worth noting that the choice of a single stimulation site was dictated by the fact that the tDCS devices only have one active electrode (25 cm^2^ surface). By contrast, using a multichannel tDCS device enabled us to stimulate a larger network of areas and target reading processing more effectively and focally. In fact, one of the main limitations of the conventional two-electrode tDCS is that the electrical field spreads distally from the stimulated area, with obvious inter-subject differences attributable to anatomical variability. On the contrary, it has been demonstrated that multi-array tDCS is effective in maximizing intensity and focality, resulting in reduced inter-subject variability [23]. With respect to the specific needs of this study, it is first important to note that reading requires the integration of various specialized brain regions within a complex neural network involved in language, visual perception, and attention [24]. Among these, the neuroimaging literature [25,26,27,28] has consistently highlighted the involvement of three key areas in the reading processes. The left inferior frontal cortex, which has been associated with the storage and sequencing of phonological information; the left temporo-parietal cortex (TPC), which is crucial for phonological decoding, as it supports grapheme-to-phoneme conversion [27,28]; and the left ventral occipito-temporal cortex (vOTC), which plays a key role in orthographic encoding and the rapid recognition of written words [29,30]. More recent findings suggest the involvement of the left posterior parietal cortex as well, potentially linked to executive control and multisensory integration during reading tasks [31,32]. Functional neuroimaging studies have also documented differences in cortical activation across age groups and clinical profiles, revealing distinct patterns among children at risk of dyslexia, children with dyslexia, and adults with reading disorders [27,33,34,35]. In particular, hypoactivation of the left TPC has been reported in children at risk of dyslexia, alongside slight reductions in the vOTC and cerebellum. In children with dyslexia, hypoactivation extended to the left inferior frontal cortex, reflecting deficits in phonological manipulation. In adults with dyslexia, hypoactivation was more localized to the left TPC; in children, however, it often involved bilateral regions of the inferior parietal lobule (IPL).

A meta-analysis by Linkersdörfer and colleagues [36] revealed a convergence between areas of functional hypoactivation and reduced grey matter volume, especially in the left fusiform gyrus and the IPL, both of which are critical for orthographic and phonological processing. Further studies have also emphasized the importance of the auditory cortex, highlighting that abnormalities in both the primary and secondary auditory regions are often linked to phonological impairments in dyslexia [37].

In summary, individuals with dyslexia typically show hypoactivation of the reading neural network in the left hemisphere when reading, compared to controls [32,38]. Conversely, they over-recruit the homologous contralateral areas, possibly as a compensatory response to reading difficulties [39]. This atypical activation pattern, which is observed in regions such as the right–middle temporal gyrus, the precentral gyrus, and the occipito-temporal areas is thought to represent an alternative route for processing linguistic input when the reading network in the left hemisphere is inefficient or underdeveloped [35,40,41]. Longitudinal evidence suggested that the right-hemisphere overactivation increased with task complexity in dyslexic children, reflecting a dynamic reorganization of neural circuits during development [42]. Bearing this in mind, we opted to use the multifocal tDCS to stimulate the key nodes in the reading neural network. Anodal (excitatory) current was applied to the 10/20 system sites corresponding to the aforementioned areas of the left frontal, temporal, parietal, and occipital cortex, while cathodal (inhibitory) activity targeted the contralateral sites. Repetitive tDCS sessions were administered alongside cognitive training, as this approach has been shown to be the most effective in the literature [for a review, see [43]].

## 2. Materials and Methods

**Subject:** This single-case study focused on a 9-year-old boy enrolled in the third grade of primary school. The child, whom we will call Harry, arrived at our clinical and research centre due to learning difficulties. A neuropsychological assessment was conducted, and the child was diagnosed with a severe mixed-type specific learning disorder in the presence of cognitive abilities in the normal range (IQ = 93; Global Ability Index = 115). Following the diagnostic evaluation and consultation with the family, it was decided to proceed with a 10-week treatment programme combining multifocal tDCS and cognitive training, with 3 sessions per week for a total of 30 sessions. The parents gave their written informed consent, and the study was conducted in accordance with the ethical standards of the Declaration of Helsinki [44]. All data collected during the study was anonymized and used exclusively for scientific research purposes. Any images, recordings, or visual representations were edited to ensure that participants could not be identified. No personal information (such as names, faces or recognizable voices) was included in publications or presentations of results. The data were stored securely and are accessible only to authorized members of the research team.

**Cognitive Training:** Depending on the stage of treatment, we used either a card game created in our laboratory for the single grapheme recognition task or a computer-based tachistoscope. The tachistoscope exercise involved presenting Italian syllables and two-syllable words in a random sequence. A fixation point appeared in the centre of the screen for 250 ms, followed by the target stimulus (a syllable or word) for a variable dura-tion according to Harry’s performance (see the Procedure section). Once the target had disappeared, a visual mask was displayed to eliminate visual persistence, thereby providing greater control over stimulus presentation. 

The exposure time of the stimuli was adjusted according to the participant’s reading ability, and performance was recorded in terms of correct responses, omissions, and self-corrections. Additionally, we calculated a trade-off between exposure time and correct responses for each day of training, providing a single performance measure that considers the ratio between these two parameters.

**Figure 1:**
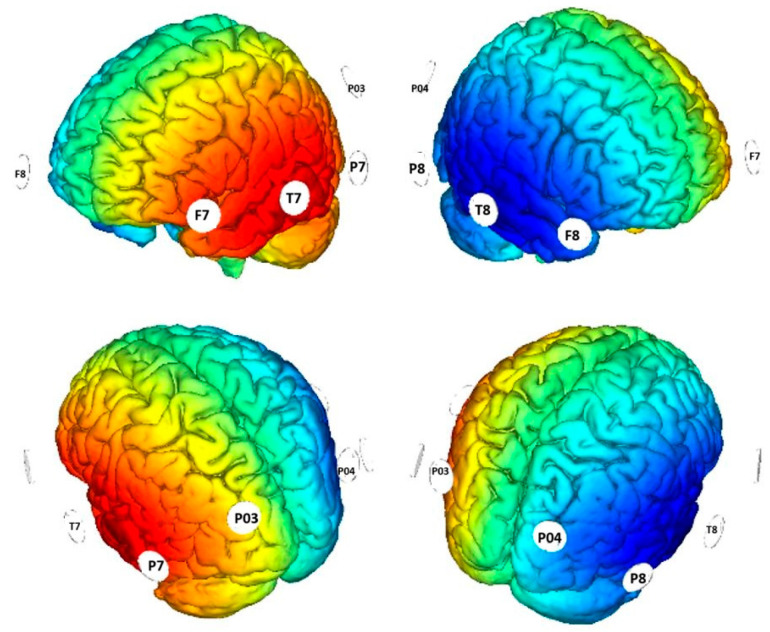
The figure shows the simulation of the cortical distribution of the applied electric field.

**Multifocal tDCS:** Direct current was delivered through the Starstim multifocal tDCS system (Barcelona, Spain) [45] using eight 3.14 cm^2^ Ag/AgCl gelled electrodes placed into a neoprene cap with a total injected current of 2000 μA. Four electrodes provided a negative current (−1000 μA) over the right cerebral cortex (F8, T8, P8, P04 sites of the 10/20 EEG system), while four electrodes supplied a positive current (1000 μA) over the left cerebral cortex (F7, T7, P7, P03). The stimulation lasted for 18 min, including 30 s of ramp-up and ramp-down. The simulation of the cortical electric field distribution was carried out using NIC 2 software (version 2.0.11), as depicted in Figure 1. Potential adverse effects of tDCS were evaluated by the experimenter at the end of each session through an interview based on the questionnaire by Brunoni et al. [46]. The questionnaire, completed by the child at the end of each session, listed potential adverse effects related to tDCS, including headache, neck pain, scalp pain, tingling, itching, burning sensation, skin redness, sleepiness, difficulty concentrating, and acute mood changes. Each symptom was rated on a 4-point scale as follows: 1 = absent; 2 = mild; 3 = moderate; and 4 = severe. Across all stimulation sessions, the child reported only a mild itching sensation.

**Neuropsychological assessment:** Cognitive and learning skills were assessed using the Wechsler Intelligence Scale for Children—Fourth Edition [47] and the MT test [48], which included reading passages and single-word recognition tasks. Reading was assessed using a list of short high-frequency words taken from the ALCE test [49]. To assess grapheme recognition, we administered a single-letter reading task from the DDE-2 battery [50]. Harry correctly named only 7 out of 21 graphemes (z score = −7) within 41 s (z score = −9).

Reading passages and high-frequency short words were assessed at baseline (pre-treatment), post-treatment, and one- and six-months follow-up. To minimize any bias, the assessment and the intervention were carried out by different researchers. In other words, the evaluator was unaware of the intervention phase when conducting the evaluation.

**Figure 2:**
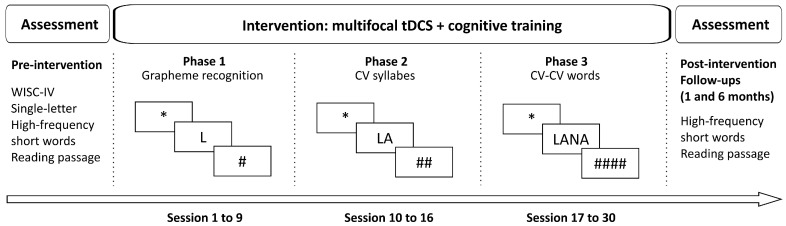
The flowchart of the experimental design. * refers to the fixation point, and # to the visual mask.

**Procedure:** After becoming familiar with the procedure, the subject was fitted with the tDCS cup, and the electrode impedance was checked. The participant was then seated in a comfortable position in front of a computer screen. In each experimental session, tDCS was delivered alongside cognitive training (online stimulation). When the stimulation stopped, so did the training. A total of 30 sessions were provided. Depending on the participant’s reading skills, three different trainings were provided, as depicted in Figure 2.

**First phase (from Session 1 to 9):** Due to Harry’s poor reading skills, only a single grapheme recognition task was provided.

**Second phase (from Session 10 to 16):** Consonant–vowel (CV) syllable training at the tachistoscope was carried out. The initial exposure time for each syllable was set to 1500 milliseconds and was gradually decreased to 600 milliseconds by the seventh day. The exposure time of the stimuli was reduced in according to the threshold for the percentage of correct responses, which was set to 70%. In particular, the performance of the last ten trials of the training session was used as a reference point: if Harry correctly named at least seven out of ten syllables, the exposure time was reduced for the next session. This approach aimed to enhance the participant’s processing speed and familiarity with the syllabic structure of the language.

**Third phase (from Session 17 to 30):** Tachistoscopic reading of bisillabic words (CV-CV) was carried out. The stimulus exposure time started at 3000 milliseconds and was gradually reduced to 1000 milliseconds. Even in this case, the exposure time of the stimuli was reduced based on a threshold of 70% accuracy, calculated from the last ten trials of the training session. This phase was designed to further improve reading fluency and efficiency, building on the skills developed in the previous stages.

## 3. Descriptives Results

**Single Grapheme Training**

No quantifiable outcomes are available for this phase of training, which aimed to ensure that Harry was able to discriminate all the graphemes of the Italian language before moving on to the second phase of syllable training. By the end of this training phase, following nine tDCS stimulations, the typical errors made by Italian dyslexics, such as letter inversions (p/q; a/e; b/d), had disappeared.

**CV Training**

**Figure 3:**
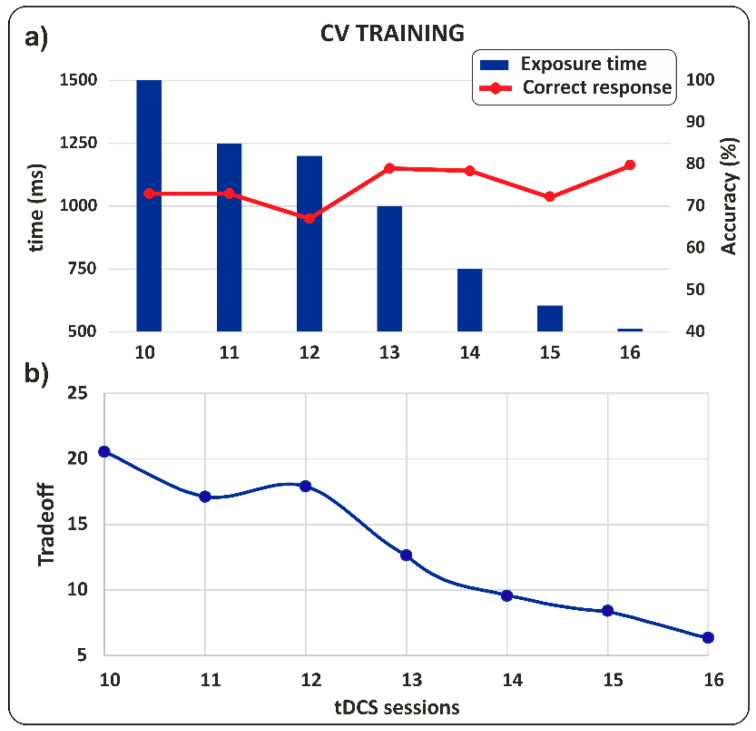
CV training. (**a**) Percentage of correct responses as a function of syllable exposure time. Note how as exposure time decreases, the percentage of correct responses increases. (**b**) Trade-off between exposure time and percentage of correct answers.

Harry’s performance showed a marked improvement during the second phase, which involved tDCS stimulation combined with tachistoscopic syllable reading training. As shown in Figure 3a, accuracy increased as the stimulus exposure time decreased. Figure 3b shows the trade-off between exposure time and the percentage of correct responses, revealing a radical shift from 20.4 (session 10) to 7.8 points (session 16). These data highlighted the effectiveness of the training in improving Harry’s reading skills in terms of both exposure and accuracy.

**CV-CV word training**

**Figure 4:**
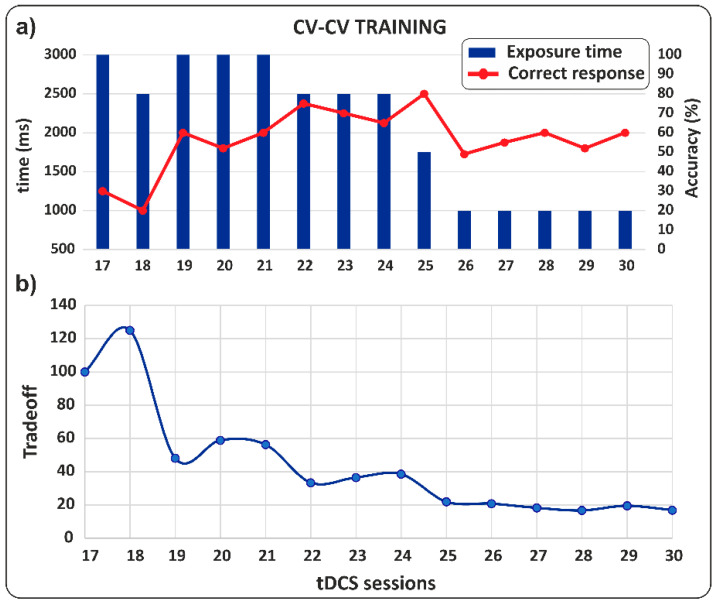
CV-CV training. (**a**) Percentage of correct responses as a function of bisyllabic word exposure time. Note how as exposure time decreases, the percentage of correct responses increases. (**b**) Trade-off between exposure time and percentage of correct responses.

Harry’s performance showed a marked improvement during the third phase, which involved tDCS stimulation combined with tachistoscopic word reading training. As shown in Figure 4a, the stimulus exposure time decreased from 3000 ms (session 17) to 1000 ms (session 30). At the same time, the percentage of correct responses increased from 30% to 65%. Figure 4b depicts the trade-off between exposure time and the percentage of correct responses, highlighting a substantial change in the ratio from 100 to 15.4 points. These findings highlighted the effectiveness of the training in enhancing Harry’s reading skills in terms of both exposure and accuracy.

**Text Reading**

**Figure 5:**
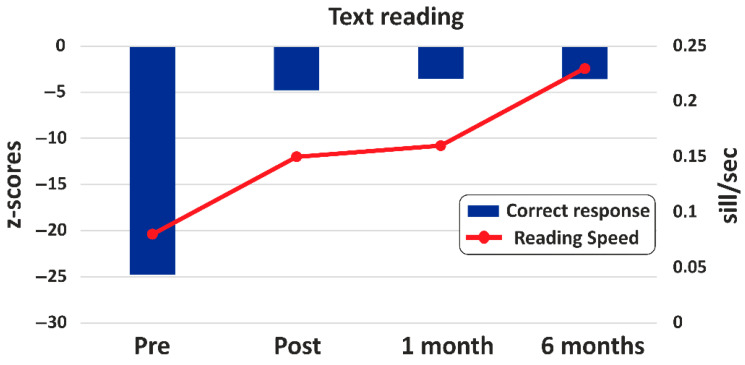
Text reading performance at the pre- and post-treatment and follow-up. Correct responses are shown in blue (z scores). The speed of reading is shown in red (syllables per second).

In terms of reading accuracy for the passage, there was a remarkable reduction in the number of errors, decreasing from 93% (−24.77 z-score) to 21% (−4.77 z-score). This improvement was sustained over time, as demonstrated by a further decrease to 16% (−3.54 z-score) at both the one- and six-month follow-up. Regarding speed, an increase was recorded from pre- (0.08 syll/s) to post-intervention (0.15 syll/s), which remained stable at the one-month follow-up (0.16 syll/s) but increased further at six months (0.23 syll/s), as shown in Figure 5 and summarized in Table 1. It is worth noting that the month with no intervention coincided with the summer school holidays. During this time, Harry received no cognitive stimulation whatsoever.

**High-frequency short words**

**Figure 6:**
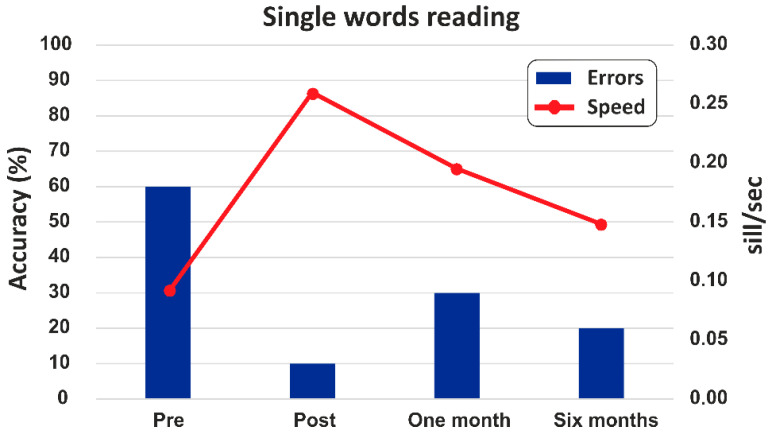
High-frequency short words. The percentages of correct responses are indicated in blue at the pre- and post-treatment and follow-up. Word reading speed, measured in syllables per second, is represented in red.

A reduction in error was observed from pre- to post-treatment, with a decrease from 60% to 10%. There was a slight increase in error in the first follow-up, reaching 30%, before stabilizing at 20% by the six-month follow-up (see Figure 6).

The reading speed increased from 0.09 to 0.26 syllables per second following treatment. At the one- and six-month follow-up, values of 0.20 and 0.15 syllables per second were recorded, respectively. See Table 1 for a summary of the results.

## 4. Comments

The present findings documented the effects of an innovative treatment for dyslexia that combines multifocal tDCS and cognitive training. Due to Harry’s severe learning difficulties, a three-step training programme was devised. Overall, the training led to remarkable improvements in terms of the accuracy of reading. When he arrived at the local service, Harry was unable to distinguish between single graphemes, but he was able to do so after undergoing nine tDCS stimulations combined with a single grapheme recognition task (phase 1). In the second stage, Harry underwent seven CV training sessions. During these sessions, the stimulus exposure time decreased from 1500 ms to 600 ms, and the error rate decreased accordingly. Then, from sessions 17 to 30, he undertook more complex CV-CV training. This enabled him to reduce the stimulus exposure time by a third (from 3000 to 1000 ms) while improving accuracy.

The treatment resulted in a remarkable enhancement in accuracy for single words (from 60% to 10% of errors) and for text reading (93% to 21% of errors), corresponding to improvements of 83% and 77%, respectively. The one-month follow-up demonstrated the stable effects of accuracy, suggesting that the skills acquired through the intervention were durable and that the cognitive gains achieved were likely to be retained in the short term. As for speed, an increase of 0.15 syllables per second was observed in text reading over six months. This equalled the annual average increase in reading speed among Italians with dyslexia (0.3 syllables per second) [5], excluding the effect of tDCS. However, from an ecological point of view, it is preferable to prioritize accuracy over speed, since the latter can be improved by adopting a more effective reading approach. It is not a case where in transparent orthographies such as Italian, children achieve high reading accuracy very rapidly, while the development of reading speed occurs more gradually over time [51]. Overall, Harry’s performance suggested that he was not compromising correctness for speed, indicating balanced skill development. In other words, the data provided a robust indication of the effectiveness of the intervention in promoting skill retention. Maintaining low error rates over time is, in fact, crucial for reinforcing the foundations of reading competence.

## 5. General Discussion

The findings of this study contribute to the growing body of evidence supporting the efficacy of the tDCS as an intervention for SLDs, particularly with regard to reading improvement. SSLDs are characterized by a complex interplay of neurodevelopmental factors, often leading to notable challenges in various areas of life [52]. Due to the chronic nature of these disorders and variability in responses to conventional rehabilitation strategies, exploring alternative interventions such as tDCS is crucial.

In our study, we used innovative multifocal tDCS technology to stimulate the key areas of the reading network in the left hemisphere and inhibit the contralateral areas. This montage, in combination with cognitive training, elicited remarkable changes in reading in a child with a diagnosis of severe dyslexia. These findings are consistent with the growing body of literature suggesting that tDCS can modulate cortical excitability and facilitate neuroplasticity, thereby promoting more effective learning pathways in individuals with SLDs [13]. If this has already been proposed by studies adopting conventional tDCS, the present findings could suggest that the effects are even more pronounced when multiarray tDCS is adopted. The neurophysiological mechanisms behind this proposal could be twofold. Firstly, the higher number of electrodes allows for critical nodes to be stimulated simultaneously. Secondly, this approach has been shown to maximize the intensity and focality of stimulation compared to the conventional tDCS apparatus [23].

Previous studies have suggested that repeated tDCS sessions, when combined with reading interventions, can effectively improve reading skills. Studies adopting the conventional two-electrode tDCS indicated that left anodal/right cathodal stimulation of the TPC improves reading pseudo- and low-frequency words [for a review, see [43]]. However, these data have limited clinical relevance, as they did not affect text reading, resulting in a lack of ecological validity. In addition, no follow-up data were presented, which casts doubt on the durability of the effects over time. By contrast, the present study demonstrated improvements in the ability to read high-frequency short words (accuracy and speed), as well as an improvement in the ability to read a passage, with stable effects at the one- and six-month follow-up. These promising findings underscored the potential for multifocal tDCS to be integrated into a comprehensive rehabilitation framework for students with SLDs. This approach could provide a multi-modal strategy that combines cognitive training with brain stimulation. In this framework, the effectiveness of tech-based neurocognitive interventions has also been demonstrated for other neurodevelopmental disorders such as ADHD [53]. These kinds of rehabilitative approaches would provide a more holistic intervention addressing both the cognitive and neurobiological aspects of learning disorders [8] in a non-invasive and operator-independent way. Another factor to consider with these approaches is the possible gain in metacognition and motivation, thereby positively impacting learning [54].

### Limitations

One of the main limitations of this research is its focus on a single, severe case. The severity of the condition made it difficult to find other patients with similar clinical features. Furthermore, due to the single-case study methodology, no statistical analysis was carried out. The absence of quantitative analysis means that the results should be treated with caution. In addition, the absence of a control group did not allow us to distinguish the contribution of the tDCS from that of the cognitive training, and it is also crucial to note that the response to treatment may vary significantly depending on the starting level of the disorder. However, for a comparison between tDCS and other dyslexia treatments, we could refer to the study by Costanzo and colleagues [15], in which an active treatment (tDCS + cognitive training to improve reading speed and phonics skills) was compared to a control condition (sham tDCS + cognitive training). The study demonstrated that only the active group received long-lasting benefits in reading, while no differences emerged in the sham group. These findings could be seen as an indirect support for the greater efficacy of tDCS compared to common cognitive interventions for dyslexia, as well as for the need to explore new approaches to treating SLDs.

It is also possible to hypothesize that the observed improvements may be partly influenced by the novelty or motivational effects associated with device-based interventions [54]. The perceived technological sophistication of tDCS may enhance participant engagement or expectations, especially in pediatric populations, potentially contributing to non-specific treatment effects. Future studies should consider variables such as the individual differences related to the severity of SLDs as well as comorbidities, in order to identify potential predictors of treatment response. Moreover, future research in the field would require larger and controlled trials to better isolate intervention-specific effects from non-specific factors. These aspects are essential to develop more personalized and effective approaches in the treatment of dyslexia.

## Figures and Tables

**Table 1:** Harry’s reading performance across different assessments from pre-treatment to the six-month follow-up. Accuracy and speed values are expressed as a percentage of error and syllables per second, respectively.

Task	Index of Performance	Pre-Treatment	Post-Treatment	One-Month Follow-Up	Six-Month Follow-Up
High-frequency short words	Accuracy	60	10	30	20
	Speed	0.09	0.26	0.20	0.15
Text reading	Accuracy	93	21	16	16
	Speed	0.08	0.15	0.16	0.23

## Data Availability

Data are available upon request.
